# Supplementary material for: Tension activation of mechanosensitive two-pore domain K+ channels TRAAK, TREK-1, and TREK-2
Source: Nat Commun. 2024 Apr 11;15:3142. doi: 10.1038/s41467-024-47208-5 (PMC11009253; doi:10.1038/s41467-024-47208-5)
Supplement: Supplementary file 1 — Supplementary Information [file 41467_2024_47208_MOESM1_ESM.pdf]

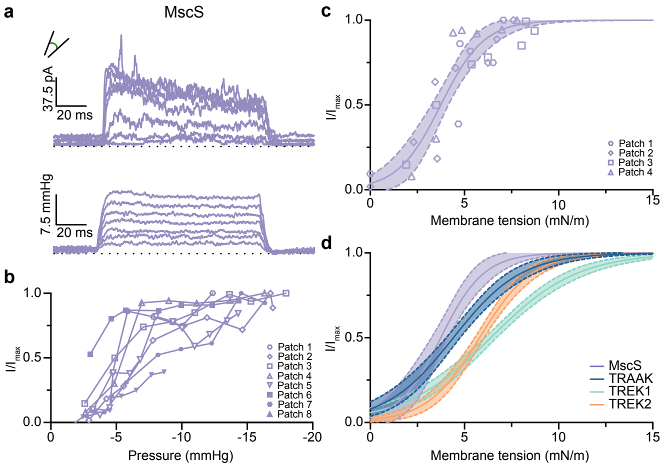

Supplementary Figure 1. Quantification of MscS activation by membrane tension.
(a) Macroscopic currents (upper) recorded at −60 mV from a MscS-containing patch in response to a pressure step protocol (lower). **(b)** Normalized current-pressure relationships from 8 MscS-containing patches. **(c)** Normalized current-tension relationships for MscS. Global fits to a Boltzmann sigmoidal with 95% confidence intervals are shown (τ_{50} = 3.7 ± 0.2 mN/m, mean ± SEM, n = 4 patches from n=3 cells). **(d)** Overlaid fits comparing tension response of MscS and mechanosensitive K2Ps.

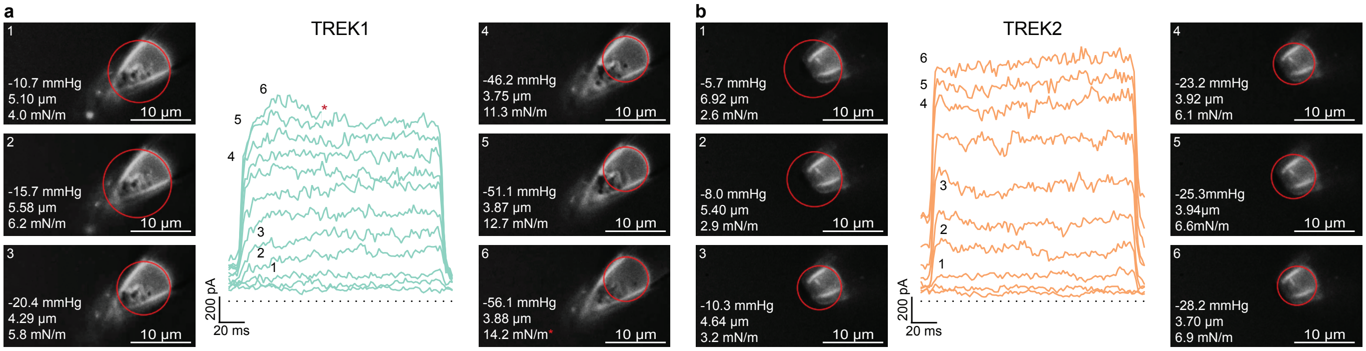

Supplementary Figure 2. Quantification of TREK-1 and TREK-2 activation by membrane tension.
Macroscopic currents (center) recorded at 0 mV from a **(a)** TREK-1- and **(b)** TREK-2-containing patch in response to a pressure step protocol. Fluorescent images of the patched membrane at the time of maximum current response during each of six pressure steps are shown. Measured pressure, patch radius (from red fit circle), and calculated tensions are shown in each image.

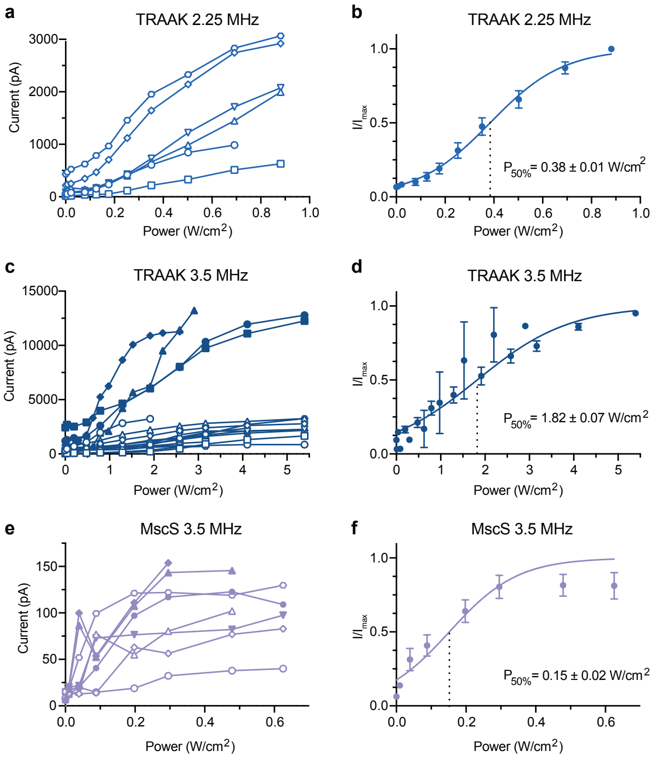

Supplementary Figure 3. Quantification of TRAAK and MscS activation by ultrasound. **(a)** Measured TRAAK currents and **(b)** normalized activation (I/I_{max}) versus 2.25 MHz ultrasound power (n = 6 patches). **(c)** Measured TRAAK currents and **(d)** normalized activation (I/I_{max}) versus 3.5 MHz ultrasound power (n = 17 patches). **(e)** Measured MscS current and **(d)** normalized activation (I/I_{max}) versus 3.5 MHz ultrasound power (n = 8 patches). Data from each patch are shown with different shapes and shading in **(a,c,e)**.

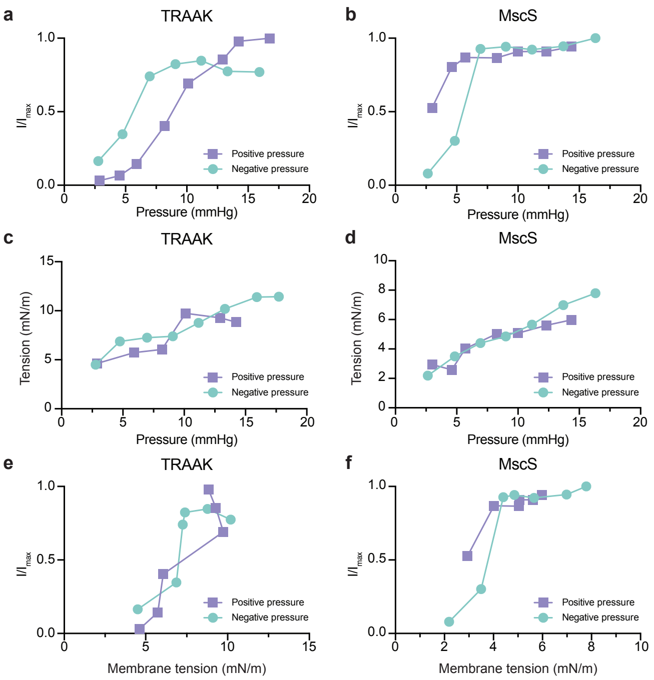

Supplementary Figure 4. TRAAK and MscS are equivalently activated by tension generated by positive and negative pressure.
Normalized current-pressure relationship for positive (periwinkle) and negative (green) pressure application for a **(a)** TRAAK-containing and **(b)** MscS-containing patch. **(c,d)** Tension-pressure relationship from data in **(a,b)**. **(e,f)** Normalized current-tension relationship for channels activated by positive (periwinkle) and negative (green) pressure.

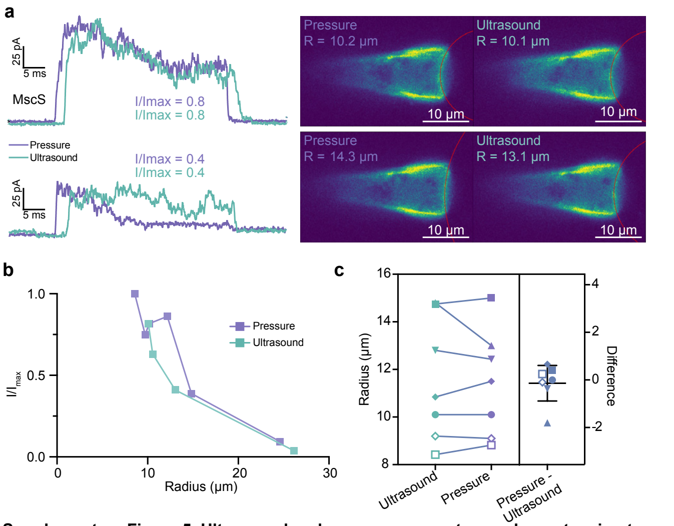

Supplementary Figure 5. Ultrasound and pressure generate membrane tension to activate MscS channels.
(a) (left) Overlaid macroscopic currents and (right) corresponding fluorescent images from a MscS-containing patch in response to pressure (periwinkle) and ultrasound (green) stimulation. Traces and images are compared from the same patch activated to high (upper) and moderate (lower) I/I_{max}. Circle fits (red) and corresponding membrane radii of curvature are indicated on the images. Similar patch radii are observed when ultrasound or pressure stimuli generate similar channel activation. **(b)** Normalized current-patch radius relationship calculated from a single patch in response ultrasound and pressure stimulation. **(c)** Comparison of patch radii observed during ultrasound or pressure stimulation that resulted in I/Imax values near 0.5. Data from 7 paired records from 5 different patches is shown ($p = 0.95$, two-tailed paired t-test, not significant).

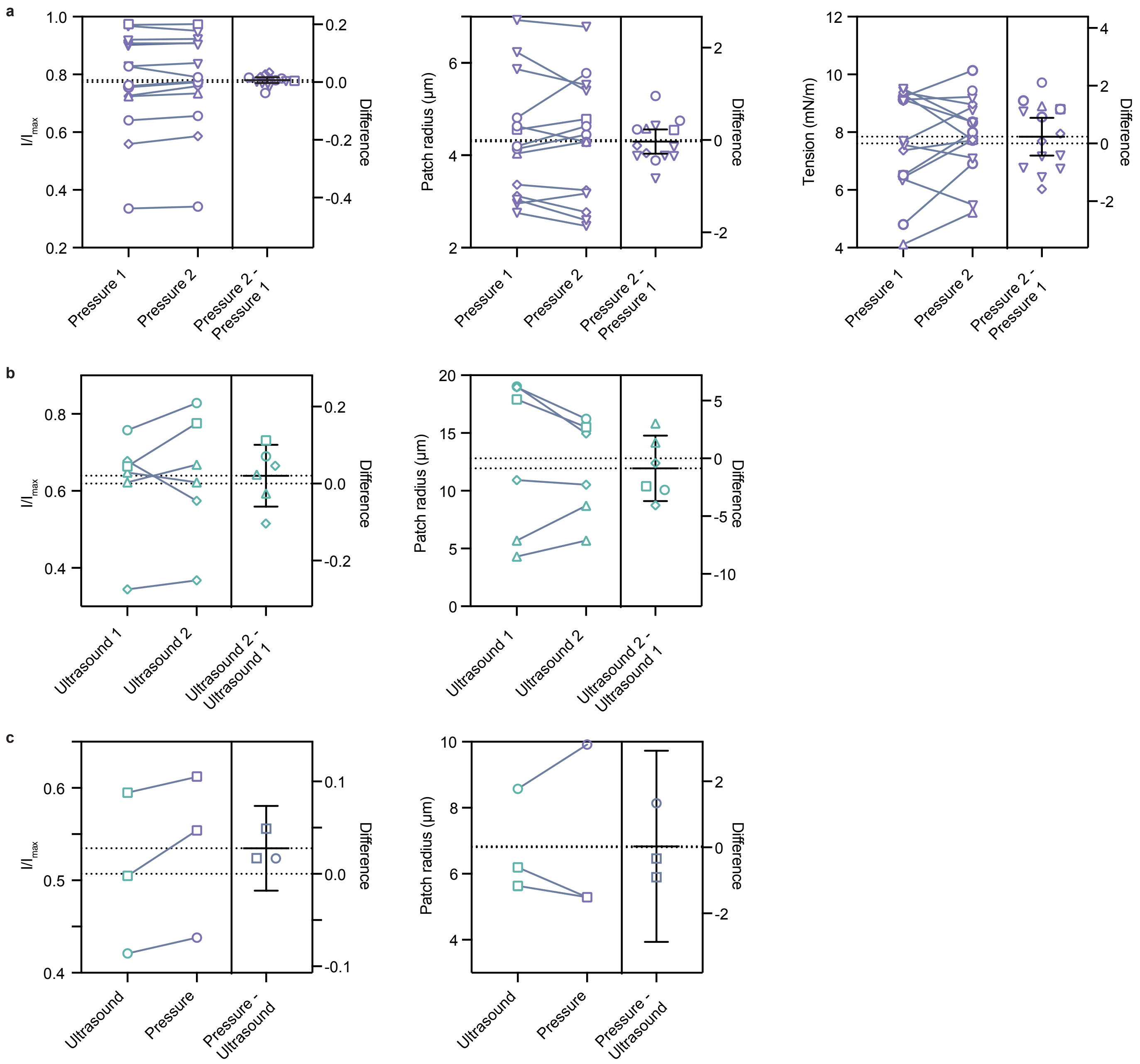

Supplementary Figure 6: Comparison of patch radius during paired stimuli that similarly activate TRAAK.
(a) Pairs of pressure stimuli from the same patch that yield comparable channel activation (left, $p = 0.17$, two-tailed paired t-test, $n=9$ paired recordings from 5 patches) show no significant difference in patch radii (center, $p = 0.79$, two-tailed paired t-test) or membrane tension (right, $p = 0.45$, two-tailed paired t-test). Each pair of records is connected by a line and each shape corresponds to a different patch. **(b)** Pairs of ultrasound stimuli from the same patch that yield comparable channel activation (left, $p = 0.54$, two-tailed paired t-test, $n=6$ paired recordings from 4 patches) show no significant difference in patch radii (center, $p = 0.47$, two-tailed paired t-test). **(c)** Pairs of stimuli (with ultrasound preceding pressure) that yield comparable channel activation (left, $p = 0.12$, two-tailed paired t-test, $n=3$ paired recordings from 2 patches) show no significant difference in patch radii (center, $p = 0.97$, two-tailed paired t-test).
